# PAP_NER: A large-scale vietnamese administrative named entity recognition corpus and hybrid deep learning architecture

**DOI:** 10.1371/journal.pone.0353166

**Published:** 2026-07-27

**Authors:** Dinh-Dien La, Tien-Bang Tran, Ngoc-Huy Du, Ngoc-Hung Dang, Trung-Nghia Phung, Van-Khanh Tran

**Affiliations:** 1 Provincial Party Committee, Minh Xuan Ward, Tuyen Quang, Vietnam; 2 Hanoi University of Science and Technology, Hanoi, Vietnam; 3 Institute of Artificial Intelligence, Thai Nguyen University of Information and Communication Technology, Thai Nguyen, Vietnam; 4 Faculty of Arts and Communications, Thai Nguyen University of Information and Communication Technology, Thai Nguyen, Vietnam; Indian Institute of Technology Patna, INDIA

## Abstract

Named Entity Recognition (NER) is fundamental for automating administrative document processing in digital government systems. However, Vietnamese NLP research faces a critical infrastructure gap: existing datasets focus on generic information extraction (news, medical) rather than domain-specific administrative text. We present PAP_NER, the first large-scale, gold-standard Vietnamese administrative NER corpus comprising 162,801 sentences with 205,807 entity annotations across five entity types critical for e-Government workflows: Agency (CQ), Legal Document (VBPL), Object (ĐT), Datetime (NG), and Quantity (SL). The dataset was constructed through a rigorous human-in-the-loop annotation pipeline, achieving an inter-annotator agreement of κ = 0.85. We demonstrate PAP_NER’s value through comprehensive benchmarking of an established hybrid deep learning architecture, PhoBERT-CRF, which couples monolingual Transformer embeddings (PhoBERT) with Conditional Random Fields for structured prediction. PhoBERT-CRF achieves 97.95% Micro F1-score on the PAP_NER test set, significantly outperforming established baselines: BiLSTM+CRF (+2.01%), multilingual XLM-RoBERTa (+2.52%), and pure Transformer approaches (+0.44%). Ablation analysis reveals that the CRF layer provides statistically significant improvements for structurally complex entities (VBPL: + 0.96%, *p* < 0.05, McNemar’s test). We release PAP_NER publicly (DOI: 10.5281/zenodo.18044019) under Creative Commons BY 4.0 license to support reproducibility and enable further research in Vietnamese administrative NLP. This work establishes a foundational dataset and methodology for addressing the Vietnamese government NER gap, with implications for low-resource language NLP research.

## 1 Introduction

### 1.1 Motivation: The vietnamese administrative NER gap

Digital government transformation requires automated processing of administrative documents at scale [1]. In the field of Natural Language Processing (NLP) [2], Named Entity Recognition (NER) [3] serves as a foundational component for extracting structured information from unstructured administrative text, enabling downstream tasks such as automated document routing, compliance verification, and administrative knowledge graph construction. However, Vietnamese NLP research faces a severe dataset scarcity problem for administrative domains. Existing datasets (VLSP 2016, 2018, 2021 [4–6], and PhoNER_COVID19 [7]) focus on generic entities (Person, Location, Organization) derived from news corpora or biomedical documents. These datasets exhibit three critical mismatches with administrative requirements [8]:

1. ***Entity Type Mismatch***: VLSP focuses on generic entities (Person, Location, Organization) derived from online news corpora. The label “Organization” fails to distinguish between a private enterprise (e.g., “Công ty TNHH ABC”) and a competent state agency (e.g., “Sở Tài chính tỉnh Thái Nguyên” (Department of Finance of Thai Nguyen Province)—a distinction with legal consequences in administrative routing. A document routed to a private company instead of the Department of Finance violates procedural law.
2. ***Structural Incompatibility***: PhoNER_COVID19 targets medical brevity with entity types like Patient, Symptom, and Medication—typically short spans (1–3 tokens). In contrast, administrative procedures contain lengthy legal citations (VBPL entities) spanning 15–20 tokens with nested hierarchical dependencies. Models trained on short-span entities demonstrate poor generalization to multi-clause legal references.
3. ***Linguistic Domain Gap***: News text (VLSP corpus source) employs journalistic language optimized for reader engagement, with varied vocabulary and informal phrasing. Administrative procedures use legal formalism—standardized terminology, passive voice constructions, and archaic compound words, e.g., “thẩm quyền giải quyết” (settlement authority), that rarely appear in news text. This vocabulary mismatch degrades transfer learning effectiveness.

To address this gap, we introduce PAP_NER (Public Administration Procedures NER), the first large-scale, domain-specific Vietnamese NER corpus engineered for administrative document processing.

### 1.2 The vietnamese administrative text challenge: A linguistic problem

Vietnamese administrative procedures exhibit a unique domain paradox that exacerbates the engineering challenge. On the one hand, these documents adhere to high-normativity, rigid, template-driven syntactic structures designed to ensure legal consistency and transparency. Standard phrases like “Hồ sơ bao gồm” (The dossier includes), “Thời gian giải quyết” (Processing time), and “Cơ quan có thẩm quyền giải quyết” (Competent authority for settlement) appear with near-deterministic regularity, suggesting that rule-based systems should suffice.

However, embedded within these rigid templates are structurally complex, nested entities that defy simple pattern matching:

1. *Challenge 1: Long Legal Citations with Nested Alphanumeric Patterns*. Vietnamese legal documents (entity type: VBPL) exhibit hierarchical citation structures such as “Nghị định 34/2016/NĐ-CP quy định chi tiết và biện pháp thi hành Luật ban hành văn bản quy phạm pháp luật” (Decree No. 34/2016/NĐ-CP detailing and providing measures for the implementation of the Law on Promulgation of Legislative Documents). These citations span 15–30 tokens, contain multiple sub-components (decree type, number, year, issuing code, date, issuing agency, subject matter), and must be extracted as atomic entities for legal traceability. Token-level classification models frequently fragment these citations—predicting the decree number correctly but missing the issuing agency or date—producing structurally invalid outputs.
2. *Challenge 2: Ambiguous Agency Names with Hierarchical Structures*. Government agencies (entity type: CQ) in Vietnam follow hierarchical naming conventions that create boundary detection ambiguity. For example, “Ủy ban nhân dân tỉnh Thái Nguyên” (Thai Nguyen Provincial People’s Committee) versus “Ủy ban nhân dân xã Hồng Tiến, tỉnh Thái Nguyên” (Hong Tien Commune People’s Committee, Thai Nguyen Province). The latter spans 10 tokens with nested geographic qualifiers. Standard Transformer models [9], using token-level Softmax classification, often truncate at the first administrative level (“Ủy ban nhân dân”), missing the critical jurisdictional qualifiers that determine routing destinations.
3. *Challenge 3: Contextual Disambiguation of Generic Terms*. The entity type “Object” (ĐT)—referring to citizens or organizations subject to administrative procedures—frequently overlaps with common nouns. The phrase “cá nhân” (individual) might refer to a procedural subject (entity) in one context but serve as a generic noun in another (“đối với cá nhân có nhu cầu” (for individuals who need)). Purely contextual embeddings from Transformers capture semantic nuance but lack the mechanism to enforce consistency across entity mentions within a document.

This domain paradox—high normativity with complex nested entities—demands a hybrid approach. Rule-based systems excel at exploiting normativity but fail on nested structures requiring semantic understanding. Deep learning models (BERT [10], RoBERTa [11], and PhoBERT [12]) capture semantic context but struggle to enforce the structural constraints inherent to legal text. Neither paradigm alone satisfies the engineering requirements.

### 1.3 Research infrastructure gap in vietnamese government NLP

Despite the urgency, the current research landscape for NER in the Vietnamese administrative domain is primarily constrained by the absence of domain-specific training infrastructure, as existing Vietnamese NER datasets reflect a severe domain imbalance. According to a 2024 systematic review [13], 18.75% of studies in this domain fail to release their datasets publicly, and research gravitates toward domains with existing resources: Public Health (12 studies) due to the COVID-19 pandemic, while core administrative functions remain underexplored—e-Government (4 studies), Human Resources (2 studies), and legal document processing (1 study). Geographically, Vietnam has limited representation compared to China (23 studies) and the USA (7 studies). For Vietnamese specifically, available datasets include VLSP (2016, 2018, 2021) [4–6] or PhoNER_COVID19 [7].

There exists limited rigorous research into **hybrid architectures** that systematically fuse the complementary strengths of these paradigms—specifically, integrating the contextual embedding power of monolingual Transformers (optimized for low-resource languages) with the probabilistic constraint mechanisms of Conditional Random Fields for normative domains. While Transformer-CRF combinations appear in general NLP literature, their specific efficacy for highly normative, low-resource languages under deployment constraints remains underexplored.

### 1.4 Contributions of this work

The central contribution of this study is a new dataset and an accompanying benchmark for Vietnamese administrative NER. Concretely, we make three contributions:

1. ***Domain-Specific Training Infrastructure (PAP_NER)***: We construct and release PAP_NER (Public Administration Procedures NER), a gold-standard corpus of 162,801 sentences with 205,807 entity annotations derived from real-world administrative procedures published by Vietnam’s National Public Service Portal and provincial government databases. Unlike existing Vietnamese datasets, PAP_NER features a fine-grained labeling scheme engineered specifically for e-Government workflows.
2. ***Comprehensive Deployment-Aware Benchmark***: Using PAP_NER, we benchmark five established architectures (BiLSTM+CRF [14], Electra [15], XLM-RoBERTa [16], PhoBERT (Softmax) [12], and PhoBERT-CRF [17]) under realistic e-Government deployment criteria—extraction accuracy (F1-score), inference latency, and model footprint—subject to operational constraints including a 4GB memory budget, sub-50ms latency per sentence, and data sovereignty requirements for government systems. The benchmark establishes strong reference baselines on the corpus and shows that the well-established PhoBERT-CRF coupling offers the best accuracy–efficiency balance: it attains the highest accuracy (97.95% F1) among architectures satisfying all constraints, while adding only 1.6 ms latency over the base PhoBERT model (Section 5.9). Rather than proposing a new architecture, we contribute a reproducible, deployment-aware evaluation protocol that practitioners can reuse to select models for low-resource government NLP in other national and administrative contexts.
3. ***Reproducibility Contribution***: We provide complete experimental documentation and dataset release, enabling community validation and extension of this work. This supports broader open science goals in Vietnamese NLP research.

## 2 Related work

### 2.1 Evolution of neural architectures for named entity recognition

The evolution of Named Entity Recognition has transitioned from statistical approaches to deep neural architectures. Early neural models, such as the BiLSTM+CRF framework [14], became the de facto standard for administrative text processing. From an engineering perspective, BiLSTM+CRF remains relevant due to its low computational footprint and its ability to enforce global constraints via the Conditional Random Field (CRF) layer [18]. However, these recurrent architectures suffer from sequential processing bottlenecks and struggle to capture the long-range semantic dependencies required to resolve nested legal citations in Vietnamese administrative documents.

The advent of Transformer-based models, such as BERT [10] and RoBERTa [19], revolutionized the field by introducing self-attention mechanisms that enable parallelized processing and deep contextual embeddings. However, standard Transformer architectures typically utilize a simple Softmax classification head (token-level classification). While sufficient for general domains, this approach often fails in normative domains (e.g., legal or biomedical), where output labels must adhere to strict state-transition rules. Consequently, recent engineering research has shifted toward Hybrid Architectures [20–22] that fuse pre-trained encoders with probabilistic decoding layers to balance semantic understanding with structural validity.

Recent work has further explored NER for low-resource and multilingual settings. Ahmad et al. [23] proposed a GPT-based approach for Urdu-English NER, demonstrating the potential of large language models for cross-lingual entity extraction. Similarly, Ullah et al. [24] applied XLM-RoBERTa to Urdu educational texts, providing insights into fine-tuning multilingual Transformers for domain-specific NER in resource-constrained languages. These studies underscore the growing interest in adapting state-of-the-art architectures to underrepresented languages and specialized domains—a challenge directly addressed by our work for Vietnamese administrative text.

### 2.2 Vietnamese language models and NLP resources

Vietnamese NLP has benefited from monolingual pretrained models. PhoBERT, trained on 20GB of Vietnamese Wikipedia and news text, provides 64k-token vocabulary coverage compared to multilingual XLM-RoBERTa’s 2.5k-token Vietnamese coverage—a 25-fold difference. This vocabulary advantage is particularly significant for domain-specific terminology in administrative text. Existing Vietnamese NER datasets include VLSP (2016, 2018, 2021) generic entities from news, and PhoNER_COVID19 (medical domain). These datasets address different linguistic domains but lack administrative specificity and scale appropriate for government applications. This study specifically targets the optimization of monolingual discriminative Transformers coupled with structured prediction layers, hypothesizing that a domain-optimized PhoBERT-CRF architecture represents the optimal intersection of accuracy, privacy, and efficiency for Vietnamese e-Government applications [25].

### 2.3 Domain-specific datasets for government NER

In computational systems engineering, high-quality training data is as critical as algorithmic design. The development of Vietnamese NER has been driven by datasets such as VLSP (2016, 2018, 2021) [4–6] and PhoNER_COVID19 [7]. However, these resources exhibit a significant “*Domain Gap*” for administrative engineering applications:

1. *Domain Mismatch for Administrative Text*: VLSP focuses on generic entities (Person, Location, Organization) derived from online news. The “Organization” label conflates legally distinct entities—private enterprises (“Công ty Cổ phần Viettel”), non-governmental organizations (“Hội Chữ thập đỏ Việt Nam”), and competent state agencies (“Ủy ban nhân dân tỉnh Hà Giang”). In administrative document routing, these distinctions carry legal weight: a citizen complaint must be processed by the competent state agency with jurisdictional authority, not forwarded to private companies.
2. *Structural Incompatibility*: PhoNER_COVID19 targets medical brevity (Patient, Symptom). In contrast, public administration procedures involve lengthy, nested legal citations (VBPL) that require models to resolve hierarchical dependencies spanning multiple clauses.

To support the training of robust discriminative architectures, this study introduces PAP_NER, a new gold-standard material specifically engineered to capture the normativity and recursive structure of administrative workflows.

### 2.4 Positioning PAP_NER in the government NER landscape

According to Ramdhani’s systematic review of Government NER research [13], the field exhibits severe domain and geographic imbalances: *Domain Distribution*: Public Health (12 studies) overwhelmingly dominates due to COVID-19 pandemic urgency, while core administrative functions remain underexplored—e-Government (4 studies), Human Resources (2 studies), Legal/Justice (1 study); *Geographic Distribution*: Research is heavily concentrated in high-resource regions—China (23 studies), USA (7 studies), India (6 studies); and *Dataset Availability Crisis*: 18.75% of Government NER studies fail to release their datasets publicly, creating reproducibility failures and forcing redundant annotation efforts. PAP_NER addresses these gaps through three design principles:

- Domain Specificity: Five entity types engineered for administrative workflow automation rather than generic information extraction.
- Scale and Quality: 162,801 sentences (3.2× larger than VLSP, 4.6× larger than PhoNER_COVID19) with gold-standard annotation quality (κ=0.85 Inter-Annotator Agreement).
- Open Access Commitment: To promote reproducibility and further research in low-resource administrative NLP, the PAP_NER dataset is made available to the research community on Zenodo (DOI: 10.5281/zenodo.18044019) under Creative Commons BY 4.0 license.

## 3 Materials and data: The PAP_NER corpus

To support the development of NER systems for Vietnamese e-Government, we engineered PAP_NER, a domain-specific training corpus that addresses the critical infrastructure gap identified in Section 2.3. This section details the end-to-end data engineering pipeline—from raw data acquisition through quality-assured annotation to final dataset statistics—with emphasis on the engineering decisions that enable cost-effective construction of gold-standard training data for resource-constrained government contexts.

### 3.1 Data acquisition and preprocessing pipeline

1. ***Source Data Selection and Rationale***. Raw textual data were harvested from two authoritative government sources to ensure domain authenticity and legal validity:
  - *National Public Service Portal* (https://dichvucong.gov.vn): Vietnam’s centralized e-Government platform publishing standardized administrative procedure descriptions for all 28 provinces and 6 centrally-governed cities. This source provides procedural templates following ISO 9001:2015 quality management standards, including structured fields for: legal basis citations, competent authorities, required dossiers, processing timelines, and service fees.
  - *Provincial Administrative Databases*: Direct access to procedure documentation from four North-East provinces (Ha Giang, Bac Ninh, Bac Giang, and Quang Ninh) provided via official data sharing agreements with provincial Departments of Information and Communications. These sources capture provincial variations in agency naming conventions, local legal references, and administrative terminology not present in national templates.
  This dual-source strategy addresses two engineering requirements: (1) National coverage via the centralized portal ensures the dataset represents standard administrative language applicable across Vietnam’s government hierarchy, and (2) Provincial diversity via regional databases captures linguistic variation essential for model generalization to unseen provinces during deployment.
2. ***Data Volume and Composition.*** The initial raw corpus comprised:
  - Approximately 6,800 distinct administrative procedures spanning 12 government sectors (education, healthcare, construction, business registration, land administration, transportation, social welfare, taxation, environmental protection, cultural heritage, agriculture, labor).
  - Approximately 487,000 raw sentences extracted from procedure descriptions.
3. ***Preprocessing Pipeline****: Ensuring Data Quality and Preventing Leakage*. To transform raw government documents into training-ready text, we implemented a four-stage preprocessing pipeline addressing data quality and methodological rigor:
  (a) *Noise Removal*: We utilized regular expressions (Regex) to strip HTML tags, non-standard encoding characters, and navigational boilerplates.
  (b) *Deduplication via MinHash for Template Leakage Prevention*: Administrative procedures often utilize standardized templates (forms), which pose a risk of data leakage if near-identical sentences appear in both Train and Test sets. For example, the phrase “Thời gian giải quyết: Trong thời hạn 10 ngày làm việc kể từ ngày nhận đủ hồ sơ hợp lệ” (Processing time: Within 10 working days from receipt of complete valid dossier) appears with minor variations (different day counts) in 1,547 procedures. To mitigate this, we applied MinHash with a Jaccard similarity threshold of 0.8 to remove near-duplicate sentences across the corpus.
  (c) *Source-Stratified Splitting to Prevent Template Memorization*: Even after deduplication, sentences from the same administrative procedure share structural patterns (e.g., all sentences describing required documents follow the pattern). Random train/test splitting would place structurally similar sentences in both sets, allowing models to memorize templates rather than learn entity recognition. We implemented Source-Stratified Splitting (also known as group-aware splitting): Instead of random shuffling, the dataset was partitioned by Procedure ID. This ensures that all sentences belonging to a specific administrative workflow appear exclusively in one split (Train, Dev, or Test), forcing the model to generalize to unseen procedures rather than memorizing formatting templates.
  (d) *Sentence Segmentation and Tokenization*: Vietnamese text uses spaces to separate constituent syllables (not words), creating segmentation ambiguity. The phrase “ủy ban nhân dân” (people’s committee) could be tokenized as:

- 4 syllables: [“ủy,” “ban,” “nhân,” “dân”] ← syllable-level
- 2 words: [“ủy_ban,” “nhân_dân”] ← word-level

We employed RDRSegmenter [26] from VnCoreNLP [27] for word segmentation.

### 3.2 Annotation Methodology and Quality Assurance

Data annotation was conducted using a strict Human-in-the-Loop (HITL) protocol to establish a Gold Standard benchmark. Unlike crowdsourced datasets, our protocol ensured domain accuracy:

1. ***Rule-based Pre-annotation*** (50% Automation).
  Administrative text exhibits high normativity, enabling regex-based extraction for structured entity types:
  - Develop Regex scripts to capture all Datetime (NG), Quantity (SL), and standardized Legal Documents (VBPL).
  - Utilize a dictionary/lexicon matching approach to detect common Object (ĐT), Agencies (CQ) (e.g., “UBND tỉnh” (Provincial People’s Committee), “Sở Tài chính” (Department of Finance),...).
  - Execute a Python script to generate pre-labeled JSON files.
2. ***LLM-Assisted Annotation*** (Additional 30% Automation). For sentences with incomplete rule-based coverage (complex agency names, nested entities, and ambiguous Objects), we employ few-shot prompting via commercial LLMs while satisfying data residency requirements.
  *Privacy and Compliance*: To adhere to data sovereignty regulations, all data processed by LLMs underwent strict local PII masking. Names, addresses, and identifying codes were replaced with placeholders (e.g., < PERSON > , < LOC>) via a regex script before transmission to cloud APIs. Labels were remapped to the original text locally.
  *Quality Assessment of LLM Annotations*: To quantify the reliability of LLM-assisted pre-labeling, we conducted a systematic quality assessment on a stratified sample of 5,000 sentences from the Label Studio revision logs. Of the LLM-generated labels, human annotators corrected 14.3% during the validation phase. Error distribution by entity type revealed that LLM accuracy was highest for Datetime (NG, 97.8% accuracy) and Quantity (SL, 96.2%), which follow predictable numerical patterns, while accuracy was lowest for Agency (CQ, 84.1%) and Object (ĐT, 86.7%) due to ambiguous entity boundaries and hierarchical naming conventions. The most common error type was boundary truncation (58% of corrections), followed by entity type misclassification (27%) and missed entities (15%). Regarding potential anchoring bias, we note that human annotators were instructed to treat all pre-labels as “suggestions only,” and the final inter-annotator agreement (κ = 0.85) was computed between the two independent human annotators—not between human and LLM—so pre-labeling did not inflate the reported agreement. A fully independent re-annotation study (without any access to pre-labels) on a held-out subset would further strengthen confidence; we note this as a methodological improvement for future corpus extensions.
3. ***Expert Human Validation and Gap Annotation*** (Remaining 20% workload)
  - All pre-annotations from Stages 1–2 are uploaded to https://labelstud.io/ for human verification.
  - Expert Annotation Team: The task was performed by seven Public Administration specialists familiar with Vietnamese administrative procedures. Annotators simply scan the predictions; press Enter to approve if correct, or drag the mouse to correct the boundaries if incorrect.
  - Double-Blind Labeling: Each sentence was independently annotated by at least two annotators.
  - Inter-Annotator Agreement (IAA): To quantify label consistency, we calculated Cohen’s Kappa (κ) [28], a standard measure of inter-annotator agreement that accounts for chance agreement, for which values above 0.60 indicate substantial agreement and values above 0.80 indicate strong agreement. The final corpus achieved an average κ=0.85, indicating strong agreement. A senior adjudicator resolved ambiguous instances to maintain the integrity of the ground truth.

#### Ethical Considerations.

This study exclusively analyzed publicly available administrative procedure documents published on Vietnam’s National Public Service Portal (https://dichvucong.gov.vn) and provincial government databases. No human participants were recruited, and no personal data beyond publicly published government documents were collected. The data annotation process involved professional annotators under standard employment agreements. Therefore, this research did not require ethical review board approval or participant consent under Vietnamese regulations. All personally identifiable information encountered during data processing was masked using automated PII removal scripts prior to any external processing (see LLM-Assisted Annotation above).

### 3.3 The semantic labeling scheme

We devised a fine-grained labeling scheme comprising five core entity types critical for e-Government automation in Table 1. Unlike generic NER tasks, these labels are defined by their legal function rather than just semantic category. The data is serialized in the CoNLL-2003 format using the BIO (Begin, Inside, Outside) tagging scheme. This standard format ensures direct compatibility with modern deep learning frameworks (Hugging Face, TensorFlow).

**Table 1:** Entity Definitions and Structural Characteristics.

Label	Entity Type	Definition & Structural Characteristics
**CQ**	Agency	Competent authorities empowered to settle procedures (e.g., *Ủy ban nhân dân tỉnh* (Provincial People’s Committee)). Note: This excludes private organizations, focusing strictly on state actors.
**ĐT**	Object	Subjects of the administrative procedure (Citizens, Organizations). Contextually ambiguous with generic nouns.
**VBPL**	Legal Document	Citations of legal bases. These exhibit high structural complexity with nested alphanumeric patterns (e.g., *Nghị định 34/2016/NĐ-CP*).
**NG**	Datetime	Temporal constraints defining processing deadlines (e.g., *Trong thời hạn 15 ngày* (Within 15 days)). Highly standardized.
**SL**	Quantity	Financial fees or dossier counts. Follows numerical patterns but requires distinguishing monetary values from citation numbers.

### 3.4 Dataset statistics and availability

Table 2 provides a comprehensive statistical breakdown of the PAP_NER dataset. The corpus comprises a total of 162,801 sentences, annotated with 205,807 entity mentions. The data is partitioned into Training (87.4%), Development (6.3%), and Test (6.3%) sets, with the training set containing 142,218 sentences to ensure robust model generalization. **Data Format and Column Structure.** The PAP_NER corpus is released in CoNLL-style column format. Each line contains a single token with tab-separated annotation columns: (1) *Word*: the segmented token produced by VnCoreNLP’s RDRSegmenter, (2) *POS*: part-of-speech tag (e.g., N for Noun, V for Verb), (3) *Chunk*: syntactic chunk tag in BIO format (e.g., B-NP for beginning of a Noun Phrase), and (4) *NER*: the named entity tag in BIO format (e.g., B-CQ for beginning of an Agency entity, O for tokens outside any entity). Sentences are separated by blank lines. For our experiments, only columns 1 (input) and 4 (target label) are used; POS and Chunk tags are provided as supplementary annotations for researchers wishing to incorporate syntactic features.

**Table 2:** PAP_NER Corpus Statistics (Split Distribution).

Entity Type	Train	Dev	Test	All
**CQ**	48,418	7,186	7,311	62,915
**NG**	27,642	3,984	4,021	35,647
**SL**	11,060	2,024	1,638	14,722
**VBPL**	23,827	867	828	25,522
**ĐT**	52,500	7,322	7,179	67,001
**# Entities in total**	163,447	21,383	20,977	205,807
**# Samples in total**	142,218	10,305	10,278	162,801

In terms of entity distribution as in Table 3, the dataset exhibits a natural skew reflecting the administrative domain:

- Dominant Classes: The most frequent entities are Object (ĐT: 67,001 entities, 32.6%) and Agency (CQ: 62,915 entities, 30.6%), collectively representing 63.2% of entities, which reflects the heavy presence of procedural subjects and government bodies in administrative texts.
- Minority Class: Quantity (SL: 14,722 entities, 7.2%) is underrepresented, as numerical specifications (fees, counts) appear less frequently than semantic entities.
- Temporal & Legal Context: There is a significant representation of Datetime (NG) (35,647 instances) and Legal Documents (VBPL) (25,522 instances), underscoring the importance of temporal constraints and legal citations in public administration.
- I-tag prevalence: I-VBPL is the most frequent entity tag (84,604 tokens), reflecting long legal citation spans.

**Table 3:** Detailed Token-Level Label Distribution.

Label	B-CQ	I-CQ	B-ĐT	I-ĐT	B-VBPL	I-VBPL	B-NG	I-NG	B-SL	I-SL	O
**Count**	63,408	20,373	67,047	48,346	25,522	84,604	35,647	35,647	14,722	16,564	3,292,690

## 4 PhoBERT-CRF hybrid architecture

To address the “Domain Gap” challenge identified in Section 1.2—where Vietnamese administrative text demands both deep semantic understanding (for disambiguating complex nested entities) and strict structural constraint enforcement (for ensuring valid BIO sequences)—we adopt PhoBERT-CRF, a well-established hybrid architecture that couples a Semantic Engine (monolingual Transformer for contextual embeddings) with a Structural Engine (Conditional Random Field for probabilistic sequence modeling). Transformer-CRF couplings are standard in sequence labeling [17]; our aim is not to propose a new architecture but to specify and benchmark this configuration on the PAP_NER corpus. This section formalizes the mathematical framework, architectural components, and training setup used in our experiments.

### 4.1 Problem formulation and notation

Let $𝐱=\{x_{1},x_{2},\ldots ,x_{n}\}$ denote an input sequence of *n* tokens derived from a Vietnamese administrative sentence, where each $x_{i}$ represents the *i*-th token. Let 𝒴 be the set of predefined entity tags following the BIO tagging scheme:


$$\begin{array}{c} 𝒴=\{\text{B-CQ},\text{I-CQ},\text{B-}D\text{T},\text{I-B-}D\text{T},\text{B-VBPL},\text{I-VBPL},\text{B-NG},\text{I-NG},\text{B-SL},\text{I-SL},\text{O}\} \end{array}$$

where |𝒴|=11 distinct labels. Our objective is to predict the optimal sequence of labels $y^{*}=\{y_{1}^{*},y_{2}^{*},\ldots ,y_{n}^{*}\}$, where $y_{i}^{*}\in 𝒴$, that maximizes the conditional probability:


$$\begin{array}{c} y^{*}=\underset{𝐲\in {𝒴}^{n}}{\text{arg}\max}\;P(𝐲|𝐱;\theta ) \end{array}$$

subject to the structural constraint that **y** forms a valid BIO sequence (e.g., an I-VBPL tag must be immediately preceded by either B-VBPL or I-VBPL, never by O or tags of different entity types).

**Key Engineering Challenge**: Standard classification approaches model each token’s label independently as $P(y_{i}|𝐱)$ failing to enforce structural validity. For instance, a token-wise Softmax classifier might predict the sequence [O, I-VBPL, B-CQ], which violates BIO constraints (I-VBPL cannot follow O). While post-processing heuristics can correct some violations, they are ad-hoc and may discard valid entity candidates. Our solution employs structured prediction via Conditional Random Fields to model label dependencies explicitly.

### 4.2 Architecture overview: Dual-engine design

**Fig 1:**
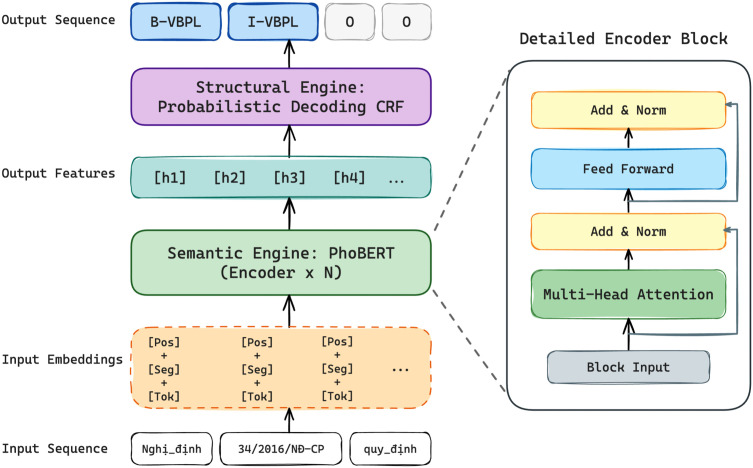
The proposed PhoBERT-CRF Hybrid Architecture for Vietnamese Administrative NER. The framework couples a Semantic Engine (PhoBERT) to capture deep contextual embeddings with a Structural Engine CRF. This dual-engine approach allows the model to leverage transfer learning for semantic understanding while strictly enforcing the probabilistic state-transition constraints required for normative legal templates. The right panel details the internal structure of the PhoBERT Transformer block used in the encoding layer.

Fig 1 presents the PhoBERT-CRF architecture which comprises three computational stages organized as a dual-engine pipeline:

1. ***Semantic Engine (PhoBERT)***: Responsible for learning deep contextual representations that capture:
  - *Lexical semantics*: Understanding that “ủy ban” (committee) + “nhân dân” (people’s) forms a compound administrative term.
  - *Long-range dependencies*: Relating “Nghị định” (decree) at position *i* to its issuing code “NĐ-CP” at position *i* + 3 within a legal citation.
  - *Contextual disambiguation*: Distinguishing “cá nhân” (individual) as an entity vs. a common noun based on surrounding procedural language.
2. Structural Engine (CRF): Responsible for enforcing sequence-level constraints:
  - BIO validity: Ensuring I-tags follow corresponding B-tags (e.g., learning P(I-VBPL∣B-VBPL)≫P(I-VBPL∣O)).
  - Entity coherence: Preventing label switching mid-entity (discouraging transitions like I-VBPL → I-CQ within a span).
  - Boundary detection: Learning domain-specific boundary patterns (e.g., legal citations often end before prepositions like “về” (about), “của” (of)).

This separation enables modular optimization: the Semantic Engine is initialized from pre-trained PhoBERT weights (transfer learning), while the Structural Engine is learned from scratch on PAP_NER’s administrative-specific transition patterns.

### 4.3 The semantic engine: PhoBERT contextualized embeddings

#### 4.3.1 PhoBERT pre-training and architectural specifications.

PhoBERT is a Vietnamese-specific RoBERTa-based language model pre-trained on 20GB of Vietnamese text from Wikipedia and news corpora.

For the encoding layer, we utilize PhoBERT, a monolingual Transformer based on the RoBERTa architecture. From an engineering perspective, PhoBERT is superior to multilingual models or generic XLM-R for this application due to domain-specific optimizations:

1. PhoBERT vs. Multilingual Models (XLM-RoBERTa, mBERT): We address the limitations of multilingual architectures, specifically regarding vocabulary and model capacity. XLM-R’s broad scope across 100 languages results in a sparse representation for Vietnamese (only 2.5k tokens), whereas PhoBERT’s 64k-token vocabulary offers a 25-fold increase in coverage for administrative terms. Furthermore, PhoBERT dedicates its entire 135M parameter capacity to Vietnamese, avoiding the dilution seen in multilingual models. The benefits of this dedicated approach are evident in our empirical results (Section 5), where PhoBERT surpasses XLM-R by 2.53% F1 on the PAP_NER benchmark.
2. PhoBERT vs. Vietnamese Generative Models (PhoGPT [29], VinaLLama [30]): We select PhoBERT to address the architectural and computational limitations of generative models. PhoBERT’s bidirectional attention is better suited for NER than the causal attention of generative models, and its compact size (135M parameters) satisfies our operational requirement to run under 4GB of memory, unlike the prohibitive resource demands of 4B+ parameter LLMs.

#### 4.3.2 Input representation and embedding layer.

For an input sequence $𝐱=\{x_{1},x_{2},\ldots ,x_{n}\}$, the model first maps each token to a dense vector representation. The embedding for token $x_{i}$ is constructed as the sum of its token embedding, position embedding, and segment embedding:


$$\begin{array}{c} {𝐞}_{i}={\text{Emb}}_{tok}(x_{i})+{\text{Emb}}_{pos}(i)+{\text{Emb}}_{seg}(x_{i}) \end{array}$$

#### 4.3.3 Transformer encoding: Multi-head self-attention and feed-forward networks.

The embedding matrix $𝐄=[{𝐞}_{1},{𝐞}_{2},\ldots ,{𝐞}_{n}]\in {\mathbb{R}}^{n\times 768}$ is processed through *N* = 12 Transformer layers. Each layer *ℓ* applies two sub-modules with residual connections and layer normalization:

1. Multi-Head Self-Attention (MHSA):

*Self-attention* computes contextualized representations by attending to all positions in the sequence:


$$\begin{array}{c} \text{Attention}(𝐐,𝐊,𝐕)=\text{Softmax}\left(\frac{𝐐{𝐊}^{T}}{\sqrt{d_{k}}}\right)𝐕 \end{array}$$

where: $𝐐={𝐇}^{\left(\ell -1\right)}{𝐖}_{Q}$ (Query matrix), $𝐊={𝐇}^{\left(\ell -1\right)}{𝐖}_{K}$ (Key matrix), $𝐕={𝐇}^{\left(\ell -1\right)}{𝐖}_{V}$ (Value matrix), $d_{k}=64$ (dimension per attention head), ${𝐇}^{\left(\ell -1\right)}\in {\mathbb{R}}^{n\times 768}$ is the input from previous layer.

*Multi-head attention* parallelizes this computation across 12 heads:


$$\begin{array}{c} \text{MHSA}({𝐇}^{\left(\ell -1\right)})=\text{Concat}({\text{head}}_{1},\ldots ,{\text{head}}_{12}){𝐖}_{O} \end{array}$$

where ${\text{head}}_{h}=\text{Attention}({𝐐}_{h},{𝐊}_{h},{𝐕}_{h})$ and ${𝐖}_{O}\in {\mathbb{R}}^{768\times 768}$ projects concatenated heads back to model dimension.

2. Position-wise Feed-Forward Network (FFN): After attention, each token’s representation is independently processed through a two-layer FFN:

$$\begin{array}{c} \text{FFN}({𝐡}_{i})=\text{GELU}({𝐡}_{i}{𝐖}_{1}+{𝐛}_{1}){𝐖}_{2}+{𝐛}_{2} \end{array}$$

where: ${𝐖}_{1}\in {\mathbb{R}}^{768\times 3072}$ (expansion layer), ${𝐖}_{2}\in {\mathbb{R}}^{3072\times 768}$ (projection layer), GELU (Gaussian Error Linear Unit): GELU(x)=x·Φ(x) where Φ is the cumulative distribution function of the standard normal distribution.

**Complete Transformer Layer:**

$$\begin{array}{c} {𝐇}^{\left(\ell \right)}=\text{LayerNorm}({𝐇}^{\left(\ell -1\right)}+\text{FFN}(\text{LayerNorm}({𝐇}^{\left(\ell -1\right)}+\text{MHSA}({𝐇}^{\left(\ell -1\right)})))) \end{array}$$

After 12 layers, we obtain the final contextualized representations:


$$\begin{array}{c} 𝐇={𝐇}^{\left(12\right)}=[{𝐡}_{1},{𝐡}_{2},\ldots ,{𝐡}_{n}]\in {\mathbb{R}}^{n\times 768} \end{array}$$

where each ${𝐡}_{i}$ encodes token $x_{i}$ in the context of the entire sentence.

### 4.4 Emission score calculation: Projecting semantic features to label space

The Transformer output **H** serves as input for emission score calculation. We project each hidden state ${𝐡}_{i}$ into the label space 𝒴 using a learnable linear transformation:


$$\begin{array}{c} 𝐏=𝐇{𝐖}^{T}+𝐛 \end{array}$$

where: $𝐖\in {\mathbb{R}}^{|𝒴|\times d_{\text{model}}}={\mathbb{R}}^{11\times 768}$ is the emission weight matrix; $𝐛\in {\mathbb{R}}^{11}$ is the emission bias vector; and $𝐏\in {\mathbb{R}}^{n\times 11}$ is the emission score matrix.

The element $P_{i,y_{j}}$ represents the *unnormalized semantic fitness score* of assigning label $y_{j}\in 𝒴$ to token $x_{i}$ based solely on its contextualized representation ${𝐡}_{i}$, without considering label sequence structure.

**Interpretation of emission scores**

- **High** $P_{i,\text{B-VBPL}}$: The model believes token $x_{i}$ is semantically likely to begin a legal document citation (e.g., “Nghị định,” “Luật,” “Thông tư”).
- **High** $P_{i,\text{O}}$: The model believes token $x_{i}$ is semantically outside any entity (e.g., prepositions “tại,” “của”; verbs “nộp,” “giải quyết”).

**Why not Softmax here?** Standard token classification applies Softmax to obtain probabilities $P(y_{i}|{𝐡}_{i})=\text{Softmax}(𝐖{𝐡}_{i}+𝐛)$ and predicts ${\hat{y}}_{i}=\text{arg}\max_{y\in 𝒴}P(y|{𝐡}_{i})$ independently for each token. This approach has a critical flaw for structured prediction. *CRF layer* (next section) models transition dependencies to maintain entity coherence.

### 4.5 The structural engine: Conditional random field for sequence modeling

To enforce structural validity, we integrate a *Conditional Random Field* (CRF) layer that models the conditional probability of the entire label sequence **y** given input **x**.

#### 4.5.1 CRF score function.

The CRF defines the score of a label sequence $𝐲=\{y_{1},y_{2},\ldots ,y_{n}\}$ as the sum of emission scores (from PhoBERT) and transition scores (learned by CRF):


$$\begin{array}{c} s(𝐱,𝐲)=\sum_{i=1}^{n}\left(P_{i,y_{i}}+A_{y_{i-1},y_{i}}\right) \end{array}$$

where: $P_{i,y_{i}}$ is the emission score for assigning label $y_{i}$ to token *i* (from Section 4.4); $A_{y_{i-1},y_{i}}$ is the *transition score* from label $y_{i-1}$ to label $y_{i}$; $𝐀\in {\mathbb{R}}^{\left|𝒴|\times |𝒴\right|}={\mathbb{R}}^{11\times 11}$ is the *transition matrix*, a learnable parameter.

**Boundary conditions**: To handle the first token (which has no predecessor), we introduce a special START state: $A_{\text{START},y_{1}}$ represents the score of beginning a sequence with label *y*_1_; $A_{y_{n},\text{END}}$ represents the score of ending a sequence with label $y_{n}$.

**Transition matrix interpretation**: The transition matrix **A** encodes the “grammar” of administrative entity sequences. These transition patterns are learned automatically from data during training, not hand-coded. The CRF discovers that Vietnamese administrative text exhibits: Long entity spans (high self-transition scores for I-tags); Strict BIO validity (impossible transitions to I-tags without matching B-tags); and Domain-specific boundary conventions (legal citations often end before prepositions like “về,” “của”).

**Implementation of BIO Constraints.** Our implementation uses the standard pytorch-crf library, which learns transition scores entirely from data without explicit hard masking. To verify that structurally invalid BIO transitions do not occur in practice, we conducted a post-hoc analysis of the learned transition matrix on the trained PhoBERT-CRF model. All structurally illegal transitions (e.g., O→I-X, B-X→I-Y where X≠Y) received learned log-transition scores below −8, corresponding to probabilities below $3\times 10^{-4}$. On the full test set (10,278 sentences, approximately 334,000 tokens), Viterbi decoding produced zero structurally invalid BIO sequences. This empirically confirms that the soft-constraint formulation, given sufficient training data (142,218 sentences), converges to de facto hard constraints—the model autonomously learns that valid BIO sequences are the only sequences with meaningful probability, without requiring explicit transition masking.

#### 4.5.2 Conditional probability via softmax over all sequences.

The CRF models the conditional probability of label sequence **y** given input **x** by normalizing the exponential score over all possible label sequences:


$$\begin{array}{c} P(𝐲|𝐱;\theta )=\frac{\exp(s\left(𝐱,𝐲\right))}{\sum_{{𝐲}^{\prime }\in {𝒴}^{n}}\exp(s\left(𝐱,{𝐲}^{\prime }\right))} \end{array}$$

where: Numerator: exp(s(𝐱,𝐲)) scores the specific sequence **y**; Denominator: $\sum_{{𝐲}^{\prime }\in {𝒴}^{n}}\exp(s\left(𝐱,{𝐲}^{\prime }\right))$ is the partition function, summing over all $|𝒴{|}^{n}$ possible label sequences (e.g., $11^{50}\approx 10^{52}$ for a 50-token sentence).

**Computational challenge**: Naively enumerating all $|𝒴{|}^{n}$ sequences is intractable. The partition function is computed efficiently via the forward algorithm using dynamic programming, with complexity $O(n\cdot |𝒴{|}^{2})$.

**Forward Algorithm for Partition Function**:

Define $\alpha_{i}(y)$ as the sum of scores of all partial sequences ending at position *i* with label *y*:


$$\begin{array}{c} \alpha_{i}(y)=\log\sum_{y^{\prime }\in 𝒴}\exp(\alpha_{i-1}y^{\prime }+A_{y^{\prime },y}+P_{i,y}) \end{array}$$

*Initialization*: $\alpha_{1}(y)=A_{\text{START},y}+P_{1,y}$

*Recursion*: For i=2,…,n:


$$\begin{array}{c} \alpha_{i}(y)={\text{LogSumExp}}_{y^{\prime }\in 𝒴}\left(\alpha_{i-1}(y^{\prime })+A_{y^{\prime },y}+P_{i,y}\right) \end{array}$$

where $\text{LogSumExp}(x_{1},\ldots ,x_{k})=\log(\sum_{j=1}^{k}\exp(x_{j}))$ prevents numerical overflow.

*Partition function*:


$$\begin{array}{c} Z(𝐱)=\sum_{{𝐲}^{\prime }\in {𝒴}^{n}}\exp(s𝐱,{𝐲}^{\prime })=\sum_{y\in 𝒴}\exp(\alpha_{n}(y)+A_{y,\text{END}}) \end{array}$$

*Complexity*: $O(n\cdot |𝒴{|}^{2})=O(n\cdot 11^{2})=O(121n)$ (linear in sequence length)

### 4.6 Training objective: Negative log-likelihood loss

During training, we maximize the log-likelihood of the true label sequence ${𝐲}^{*}$ for each training example $\left(𝐱,{𝐲}^{*}\right)$:


$$\begin{array}{c} ℒ(\theta )=-\log P({𝐲}^{*}|𝐱;\theta )=-s(𝐱,{𝐲}^{*})+\log Z(𝐱) \end{array}$$

where: θ denotes all model parameters: PhoBERT weights, emission matrix **W**, emission bias **b**, and CRF transition matrix **A**; $s(𝐱,{𝐲}^{*})=\sum_{i=1}^{n}(P_{i,y_{i}^{*}}+A_{y_{i-1}^{*},y_{i}^{*}})$ is the score of the gold sequence; and logZ(𝐱) is the log-partition function (computed via forward algorithm).

**Intuition**: The loss encourages:

- Increasing $s(𝐱,{𝐲}^{*})$: Make the gold sequence score higher by: Increasing emission scores $P_{i,y_{i}^{*}}$ for correct labels; or Increasing transition scores $A_{y_{i-1}^{*},y_{i}^{*}}$ for correct label transitions.
- Decreasing logZ(𝐱): Reduce the partition function by decreasing scores of incorrect sequences.

### 4.7 Inference: Viterbi decoding for optimal sequence prediction

At test time, we seek the label sequence $\hat{𝐲}$ that maximizes the score function:


$$\begin{array}{c} \hat{𝐲}=\text{arg}\max_{𝐲\in {𝒴}^{n}}s(𝐱,𝐲)=\text{arg}\max_{𝐲\in {𝒴}^{n}}\sum_{i=1}^{n}\left(P_{i,y_{i}}+A_{y_{i-1},y_{i}}\right) \end{array}$$

Since P(𝐲∣𝐱)∝exp(s(𝐱,𝐲)) and the exponential is monotonic, maximizing score is equivalent to maximizing probability. The partition function Z(𝐱) is constant for a given input, so it cancels out in argmax operation.

**Viterbi Algorithm**: We compute $\hat{𝐲}$ efficiently using dynamic programming:

Define $\delta_{i}(y)$ as the maximum score of any partial sequence ending at position *i* with label *y*:


$$\begin{array}{c} \delta_{i}(y)=\max_{y^{\prime }\in 𝒴}\left(\delta_{i-1}(y^{\prime })+A_{y^{\prime },y}+P_{i,y}\right) \end{array}$$

The Viterbi algorithm guarantees finding the *globally optimal* label sequence under the CRF model, whereas greedy decoding (selecting $\text{arg}\max_{y}P_{i,y}$ independently for each token) can produce suboptimal or invalid sequences.

## 5 Experiments and results

To validate the efficacy of the PhoBERT-CRF framework, we conducted a comprehensive benchmarking study against established neural architectures. This section details the experimental setup, baseline configurations, and a rigorous analysis of performance across semantic, structural, and computational metrics.

### 5.1 Experimental setup and implementation details

All experiments were conducted using the PyTorch framework on a high-performance computing environment equipped with a single NVIDIA A100 GPU (40GB VRAM). To ensure reproducibility, we fixed the random seed to 42 for all initialization steps.

**Training Hyperparameters**: We employed a systematic hyperparameter search on the development set to identify optimal configurations. The final hyperparameters are in the Table 4.

**Table 4:** Hyperparameter Configuration and Optimization Strategy.

Hyperparameter	Value	Search Space Explored	Selection Criterion
Optimizer	AdamW	{Adam, AdamW, SGD}	AdamW: best dev F1 (97.82%)
Learning rate (PhoBERT)	$2\times 10^{-5}$	{$1\times 10^{-5},2\times 10^{-5},5\times 10^{-5}$}	$2\times 10^{-5}$: stable convergence
Learning rate (CRF)	$1\times 10^{-3}$	{$5\times 10^{-4},1\times 10^{-3},5\times 10^{-3}$}	$1\times 10^{-3}$: fastest CRF learning
LR scheduler	Linear decay with warmup	{Constant, Linear, Cosine}	Linear: best final performance
Warmup ratio	0.1 (10% of total steps)	{0.05, 0.1, 0.2}	0.1: prevents early instability
Batch size	32	{16, 32, 64}	32: optimal GPU utilization
Max epochs	20	{10, 20, 30}	20: early stopping typically at epoch 15–17
Early stopping patience	3 epochs	{2, 3, 5}	3: balances exploration vs. overfitting
Max sequence length	256 tokens	{128, 256, 512}	256: covers 99.7% of sentences
Weight decay	0.01	{0.001, 0.01, 0.1}	0.01: optimal regularization
Gradient clipping	Max norm 1.0	{0.5, 1.0, 5.0}	1.0: prevents gradient explosion
Dropout	0.1	{0.0, 0.1, 0.2}	0.1: balances regularization vs. capacity
Mixed precision	FP16	{FP32, FP16}	FP16: 2× training speedup, negligible accuracy loss

### 5.2 Evaluation metrics

To rigorously quantify the extraction performance of the proposed framework, we adhere to the standard CoNLL-2003 evaluation protocol. We report performance based on Precision (P), Recall (R), and F1-Score. Crucially, we utilize the Strict Matching criterion: a predicted entity is counted as a True Positive (*TP*) if and only if both the predicted boundary span and the entity category exactly match the ground truth. The metrics are calculated as follows:


$$\begin{array}{c} \text{Precision}=\frac{TP}{TP+FP}\; \end{array}$$

$$\begin{array}{c} \text{Recall}=\frac{TP}{TP+FN}\; \end{array}$$

$$\begin{array}{c} \text{F1-Score}=\frac{2\times \text{Precision}\times \text{Recall}}{\text{Precision}+\text{Recall}}\; \end{array}$$

where *TP* (True Positives) denotes entities correctly identified in both span and type; *FP* (False Positives) denotes entities incorrectly predicted or misclassified; and *FN* (False Negatives) represents ground-truth entities that the model failed to detect. The F1-Score, being the harmonic mean of Precision and Recall, serves as the primary metric for comparing architectural effectiveness.

**Aggregation schemes**:

- Micro-averaging: Aggregate TP, FP, FN across all entity types, then compute P/R/F1 (gives equal weight to each entity instance)
- Macro-averaging: Compute P/R/F1 for each entity type separately, then average (gives equal weight to each entity type)

We report Micro F1 as the primary metric (standard in NER literature) and provide per-entity-type breakdowns for fine-grained analysis.

**Evaluation modes**. Following the MUC/SemEval evaluation framework, we report four complementary evaluation modes in our detailed analysis tables:

- *Strict*: Both entity boundaries and entity type must exactly match the ground truth. This is the most demanding criterion and serves as our primary metric.
- *Partial*: Entity type must match, and the predicted span must overlap with the ground truth span (partial boundary overlap receives proportional credit).
- *Entity Type*: Only entity type correctness is evaluated, regardless of boundary accuracy. This isolates classification performance from boundary detection.
- *Exact*: Entity boundaries must exactly match, regardless of whether the entity type is correct. This isolates boundary detection from classification.

**Statistical Significance Testing** To determine whether performance differences between models are statistically significant (not due to random variation), we employ McNemar’s Test [31]—a non-parametric test for paired binary outcomes.

### 5.3 Baseline architectures and comparative framework

To establish a comprehensive comparative evaluation, we selected four baseline architectures spanning the design space from lightweight statistical models to heavy pre-trained Transformers. Our selection criteria prioritize:

- Architectural diversity: Cover recurrent (BiLSTM), discriminative Transformers (Electra, PhoBERT), multilingual Transformers (XLM-R).
- Deployment feasibility: Exclude models violating deployment constraints (e.g., PhoBERT-large, XLM-R-large exceed memory budget)
- Prior Vietnamese NER benchmarks: Include models with published results on VLSP/PhoNER datasets for contextualization.

#### 5.3.1 BiLSTM+CRF.

The model represents the classical, pre-Transformer approach to Named Entity Recognition that dominated the field between 2016 and 2018. The architecture consists of a two-layer Bidirectional LSTM encoder with 256 hidden units per direction, followed by a Conditional Random Field (CRF) decoder that learns an 11×11 transition matrix for BIO tags. For input representation, the model concatenates fixed 300-dimensional pre-trained FastText Vietnamese word embeddings with character-level features derived from randomly initialized 50-dimensional embeddings processed via a 1D CNN (kernel size 3, 64 filters), resulting in a 364-dimensional final token vector. With a total of 4.8 million trainable parameters (excluding the fixed FastText embeddings), the model was trained from scratch on the PAP_NER dataset for 50 epochs using SGD with a learning rate of 0.01 and momentum of 0.9. This baseline is included to provide a structural modeling comparison—isolating the impact of contextual embeddings by sharing the CRF component with our proposed approach—and to benchmark against a lightweight architecture suitable for deployment scenarios requiring low latency (15–20ms).

#### 5.3.2 NER-vietnamese-electra-base.

To investigate the impact of alternative pre-training objectives, we employ the NER-Vietnamese-Electra-Base model. Unlike standard Masked Language Modeling (MLM), this architecture is based on the ELECTRA framework, which utilizes a discriminative Replaced Token Detection task where the model identifies tokens replaced by a generator. We utilize the publicly available NlpHUST/ner-vietnamese-electra-base checkpoint (110M parameters), which was pre-trained on 20GB of Vietnamese Wikipedia and news data using a 30k BPE vocabulary. For our experiments, the model is fine-tuned on the PAP_NER dataset using a standard token-level Softmax classification head, intentionally excluding a CRF layer to serve as a “pure” Transformer baseline. Fine-tuning was performed for 15 epochs with a batch size of 32 and a learning rate of $3\times 10^{-5}$. This baseline allows us to compare the efficiency of discriminative pre-training against MLM-based approaches (such as PhoBERT) and isolates the contribution of structural modeling by removing the transition logic inherent in CRF-based architectures.

#### 5.3.3 XLM-RoBERTa-Base.

To evaluate the efficacy of large-scale cross-lingual transfer learning, we employ XLM-RoBERTa-Base, a model trained on 2.5TB of CommonCrawl data covering 100 languages with a Masked Language Modeling (MLM) objective. With 270 million parameters and a vocabulary of 250k subword tokens, this model is significantly larger than its monolingual counterparts (approximately 2× the size of PhoBERT). We fine-tune the standard xlm-roberta-base checkpoint on the PAP_NER dataset using a token-level Softmax classification head without a CRF layer. Due to the substantial memory footprint of the 270M parameter architecture, we reduce the batch size to 16, training for 20 epochs with a learning rate of $2\times 10^{-5}$. Inclusion of this baseline serves two primary purposes: first, to test the hypothesis that massive cross-lingual capacity can compensate for the lack of language-specific specialization; and second, to act as a boundary test for deployment feasibility, as its 1.12GB file size pushes the limits of our target 4GB memory budget.

#### 5.3.4 PhoBERT-Base (Softmax).

To rigorously isolate the contribution of the CRF layer, we establish PhoBERT-Base (Softmax) as a critical ablation baseline. This model utilizes the identical https://huggingface.co/vinai/phobert-base encoder and hyperparameter configuration as our proposed approach, but replaces the structured CRF decoder with a standard token-level Softmax classification head. Specifically, a linear layer $W\in {\mathbb{R}}^{768\times 11}$ projects the hidden states to label logits, where predictions are generated independently for each token via ${\hat{y}}_{i}={\text{argmax}}_{y\in Y}\text{Softmax}(Wh_{i}{)}_{y}$. By eliminating the transition logic while maintaining the exact same semantic engine, this baseline allows us to directly verify the hypothesis that explicit structural modeling is necessary for handling the complex label dependencies in Vietnamese administrative texts, independent of the encoder’s contextual understanding.

To ensure fair comparison across architectures with different input representations, Table 5 summarizes the segmentation and tokenization strategy used for each baseline. For models using subword tokenization (all except BiLSTM+CRF), we adopt the standard first-subword labeling strategy: the NER label is assigned to the first subword token of each word, while subsequent subword tokens receive a special X label that is ignored during loss computation and evaluation. This ensures consistent label alignment across architectures with different tokenization granularities.

**Table 5:** Input Representation and Tokenization Strategy per Baseline.

Model	Segmentation	Tokenizer	Vocab Size
BiLSTM+CRF	VnCoreNLP (word)	FastText (fixed)	10k
Electra-Base	Syllable-level	BPE	30k
XLM-R-Base	Syllable-level	SentencePiece	250k
PhoBERT / PhoBERT-CRF	VnCoreNLP (word)	BPE	64k

### 5.4 Baseline architecture comparison

Table 6 presents key comparisons of baselines. Analysis of the computational overhead reveals that the integration of the CRF layer into the PhoBERT architecture incurs a negligible cost. Specifically, PhoBERT-CRF increases the model size by only 2MB (0.4%) and adds a mere 1.6ms (3.8%) to the inference latency compared to the PhoBERT-Softmax baseline. In terms of deployment constraints, all evaluated models, with the exception of the memory-intensive XLM-RoBERTa-Base, comfortably operate within the strict 2GB inference memory budget. Furthermore, regarding real-time applicability, all baselines demonstrate high efficiency, satisfying the sub-50ms latency requirement on standard CPU hardware.

**Table 6:** Baseline Architecture Comparison.

Model	Type	Parameters	Pre-training Data	Decoder	Memory	Latency
BiLSTM+ CRF	Recurrent	4.8M	FastText embeddings (fixed)	CRF	45MB	15.2ms
Electra-Base	Transformer	110M	20GB Vietnamese (RTD)	Softmax	440MB	41.3ms
XLM-R-Base	Transformer	270M	2.5TB Multilingual (MLM)	Softmax	1.12GB	46.8ms
PhoBERT (Softmax)	Transformer	135M	20GB Vietnamese (MLM)	Softmax	540MB	42.5ms
**PhoBERT- CRF**	**Hybrid**	**135M**	**20GB Vietnamese (MLM)**	**CRF**	**542MB**	**44.1ms**

### 5.5 Overall performance analysis

Table 7 presents the comparative results. The hybrid PhoBERT+CRF model achieved the highest performance across all metrics.

- Strong Baselines Achievement: Our hybrid model reached a Micro F1-score of 97.95%, significantly outperforming the BiLSTM+CRF baseline (+2.01%) and the multilingual XLM-RoBERTa (+2.52%). This confirms that hand-crafted features in statistical models cannot compete with the deep contextual embeddings of Transformers in this domain.
- Performance of Pure Transformer Baselines: Evaluation of the pure Transformer architectures reveals a distinct performance hierarchy: PhoBERT-Softmax (97.51%) leads, followed by Electra (95.63%) and XLM-R (95.43%). This ranking validates two critical hypotheses regarding model selection for Vietnamese administrative NER. First, the results confirm a significant “monolingual advantage,” as the Vietnamese-specific PhoBERT outperforms the massive, multilingual XLM-R by 2.08% in F1-score, despite being half the size (135M vs. 270M parameters). This demonstrates that for specialized administrative domains, language-specific optimization is far more valuable than raw cross-lingual capacity. Second, regarding pre-training objectives, PhoBERT’s Masked Language Modeling (MLM) approach proves superior to Electra’s Replaced Token Detection (RTD) for this specific task, yielding a nearly 2% performance margin.
- The “Electra Anomaly”: Surprisingly, the Electra baseline underperformed (95.63%). We hypothesize that Electra’s generator-discriminator training objective is less effective than PhoBERT’s Masked Language Modeling (MLM) for capturing the specific vocabulary of Vietnamese administrative law.
- Semantic vs. Structural: The pure PhoBERT model (97.51%) is a close second, but the addition of the CRF layer in our hybrid model pushes the performance to 97.95%. This +0.44% gain is critical in the legal domain, where boundary precision is paramount.

**Table 7:** Comparative Analysis of Model Performance (Strict F1-Score, mean ± std over 3 random seeds).

Entity Type	BiLSTM+ CRF	XLM-RoBERTa	PhoBERT	PhoBERT+ CRF	NER-Viet. Electra
CQ (Agency)	96.36 ± 0.04	94.73 ± 0.04	97.56 ± 0.06	**97.75** ± 0.02	95.00 ± 0.04
ĐT (Object)	93.52 ± 0.08	93.49 ± 0.09	96.42 ± 0.03	**97.20** ± 0.02	93.51 ± 0.06
VBPL (Legal Doc)	93.91 ± 0.09	96.21 ± 0.06	96.13 ± 0.04	**97.09** ± 0.05	96.20 ± 0.06
NG (Datetime)	99.76 ± 0.01	99.16 ± 0.02	**99.99** ± 0.01	99.94 ± 0.01	99.98 ± 0.01
SL (Quantity)	96.64 ± 0.08	96.36 ± 0.16	96.86 ± 0.04	**97.64** ± 0.02	95.99 ± 0.03
**Micro F1**	95.94 ± 0.04	95.43 ± 0.01	97.51 ± 0.03	**97.95** ± 0.01	95.63 ± 0.04
**Macro F1**	96.85 ± 0.05	96.00 ± 0.05	97.41 ± 0.03	**98.22** ± 0.03	97.99 ± 0.09

### 5.6 Fine-grained entity analysis

The performance variation across specific entity tags in Table 7 highlights the “*Domain Gap*” challenges inherent to administrative texts. We analyze each entity class in order of difficulty, from the most challenging to the most regular.

#### 5.6.1 Legal documents (VBPL): The hardest challenge.

The VBPL tag represents legal citations (e.g., “Decree 34/2016/NĐ-CP”), which contain complex alphanumeric patterns and nested dependencies. Performance across models reveals a clear stratification:

- BiLSTM+CRF achieved the lowest F1 of 93.91%, reflecting the inability of static embeddings to capture the nested structure of Vietnamese legal citations.
- XLM-RoBERTa and Electra performed comparably at 96.21% and 96.20% respectively, suggesting that multilingual and discriminative pre-training both capture cross-lingual legal citation patterns to a similar degree.
- PhoBERT reached 96.13%, slightly below the multilingual baselines on this specific class—an indication that monolingual pre-training alone does not suffice for structurally complex entities without additional boundary constraints.
- PhoBERT+CRF achieved the top score of 97.09%, outperforming all baselines. The + 0.96% improvement over pure PhoBERT demonstrates that the CRF’s transition constraints successfully resolve nested citation boundaries (e.g., enforcing that a Number token follows a Document Type token) that the Softmax head fragments.

#### 5.6.2 Objects (ĐT): Resolving semantic ambiguity.

The ĐT tag (Individuals/Organizations) is the most semantically ambiguous class, as the same noun phrase can denote an administrative object or a generic reference depending on context.

- BiLSTM+CRF and Electra both struggled at 93.52% and 93.51% respectively, while XLM-RoBERTa performed worst at 93.49%. The narrow spread among these three models (0.03% range) suggests a common failure mode: without Vietnamese-specific contextual understanding, all three architectures confuse administrative roles with generic nouns at roughly the same rate.
- PhoBERT dramatically improved to 96.42% (+2.93% over BiLSTM+CRF), confirming that monolingual contextual embeddings are essential for resolving this semantic ambiguity.
- PhoBERT+CRF further raised performance to 97.20% (+0.78% over PhoBERT), indicating that the CRF’s global sequence optimization helps distinguish specific administrative roles from generic text by enforcing valid entity transition patterns.

#### 5.6.3 Agency (CQ): Context-dependent recognition.

The CQ tag identifies government agency names, which are relatively well-defined but can appear as abbreviations or in context-dependent forms.

- Electra achieved the lowest F1 at 95.00%, followed by XLM-RoBERTa at 94.73%. The poor performance of XLM-R on this class (despite stronger performance on VBPL) suggests that agency name recognition requires Vietnamese-specific knowledge rather than cross-lingual pattern matching.
- BiLSTM+CRF reached 96.36%, outperforming both Electra and XLM-R, indicating that even static embeddings with CRF constraints can capture agency name patterns when combined with character-level features.
- PhoBERT achieved 97.56% and PhoBERT+CRF reached 97.75% (+0.19%), the smallest CRF improvement among all entity classes. This confirms that agency names are primarily a semantic recognition problem where contextual embeddings dominate, with boundary constraints providing only marginal additional benefit.

#### 5.6.4 Quantity (SL): Numerical expression recognition.

The SL tag captures quantity expressions in administrative text, including numerical values, percentages, and measurement units.

- Electra achieved the lowest F1 at 95.99%, followed by XLM-RoBERTa at 96.36%. BiLSTM+CRF reached 96.64%, outperforming both Transformer baselines on this class—a notable result suggesting that the structural regularity of quantity expressions favors CRF-based boundary detection.
- PhoBERT achieved 96.86%, while PhoBERT+CRF led at 97.64% (+0.78% over PhoBERT). The relatively large CRF gain on SL indicates that quantity expressions, while structurally regular, benefit from transition constraints that ensure complete span capture (e.g., preventing fragmentation of “15 ngày làm việc” into separate tokens).

#### 5.6.5 Datetime (NG): Near-perfect recognition.

For the Datetime class, all models performed exceptionally well, with F1 scores ranging from 99.16% (XLM-RoBERTa) to 99.99% (PhoBERT).

- The uniformly high performance (≥99.16%) across all architectures confirms the high normativity of datetime expressions in Vietnamese administrative text. These entities follow rigid templates (e.g., “ngày 15 tháng 3 năm 2024”) that are trivially recognizable even by simpler models.
- PhoBERT achieved the highest F1 at 99.99%, marginally surpassing PhoBERT+CRF at 99.94%. The slight decrease with CRF (−0.05%) is consistent with the observation that structural constraints add negligible value—and may even introduce minor noise—for entities with perfectly regular patterns.
- **Implication**: For applications only requiring datetime extraction, lightweight models like BiLSTM+CRF (99.76%) are sufficient and offer a 3× latency advantage. However, for comprehensive semantic extraction across all entity types, the hybrid PhoBERT+CRF approach remains necessary.

#### 5.6.6 Robustness check: Strict vs. partial matching.

Across all models, the Partial F1 scores (which credit overlapping boundaries) are virtually identical to the Strict F1 scores (e.g., PhoBERT+CRF Strict: 97.95% vs. Partial: 97.97%).

**Analysis**: This convergence indicates that when the models predict an entity, they almost always predict the exact span correctly. The errors are rarely partial overlaps; they are mostly complete misses or type misclassifications. This characteristic is typical of high-quality, gold-standard datasets with consistent annotation guidelines.

### 5.7 Granular performance analysis across models and entity classes

To provide a more fine-grained understanding of model behavior, we present a systematic analysis of per-class Precision (P), Recall (R), and F1-score across all four architectures (Tables 8–11), complemented by the Macro F1 and Micro–Macro divergence analysis from Table 7.

**Table 8:** Detailed Performance Analysis: PhoBERT.

Tag	Strict			Partial			Entity Type			Exact		
	P	R	F1	P	R	F1	P	R	F1	P	R	F1
CQ	98.85	96.39	97.60	98.85	96.39	97.60	99.59	97.11	98.34	98.85	96.39	97.60
ĐT	96.97	95.93	96.45	96.97	95.93	96.45	99.27	98.20	98.73	96.97	95.93	96.45
VBPL	95.26	97.10	96.17	95.26	97.10	96.17	97.99	99.88	98.92	95.26	97.10	96.17
NG	99.98	100.00	99.99	99.98	100.00	99.99	99.98	100.00	99.99	99.98	100.00	99.99
SL	97.18	96.59	96.88	97.18	96.59	96.88	99.51	98.90	99.20	97.18	96.59	96.88
**Micro**	98.15	96.97	**97.55**	98.16	96.98	97.57	99.44	98.24	98.83	98.16	96.98	97.57
**Macro**	97.65	97.20	**97.42**	97.65	97.20	97.42	99.27	98.82	99.04	97.65	97.20	97.42

**Table 9:** Detailed Performance Analysis: PhoBERT+CRF.

Tag	Strict			Partial			Entity Type			Exact		
	P	R	F1	P	R	F1	P	R	F1	P	R	F1
CQ	98.92	96.65	97.77	98.92	96.65	97.77	99.44	97.15	98.28	98.92	96.65	97.77
ĐT	98.55	95.82	97.17	98.55	95.82	97.17	99.79	97.02	98.38	98.55	95.82	97.17
VBPL	96.42	97.71	97.06	96.42	97.71	97.06	98.57	99.88	99.22	96.42	97.71	97.06
NG	99.93	99.98	99.95	99.93	99.98	99.95	99.93	99.98	99.95	99.93	99.98	99.95
SL	98.15	97.20	97.67	98.15	97.20	97.67	99.63	98.66	99.14	98.15	97.20	97.67
**Micro**	98.83	97.09	**97.95**	98.85	97.10	97.97	99.60	97.85	98.72	98.85	97.10	97.97
**Macro**	98.17	98.29	**98.23**	98.17	98.29	98.23	99.38	99.50	99.44	98.17	98.29	98.23

**Table 10:** Detailed Performance Analysis: BiLSTM+CRF.

Tag	Strict			Partial			Entity Type			Exact		
	P	R	F1	P	R	F1	P	R	F1	P	R	F1
CQ	97.37	95.28	96.32	97.37	95.28	96.32	98.38	96.27	97.31	97.37	95.28	96.32
ĐT	97.35	89.97	93.51	97.35	89.97	93.51	99.61	92.06	95.69	97.35	89.97	93.51
VBPL	94.72	93.12	93.91	94.72	93.12	93.91	98.53	96.86	97.69	94.72	93.12	93.91
NG	99.98	99.55	99.76	99.98	99.55	99.76	99.98	99.55	99.76	99.98	99.55	99.76
SL	97.69	95.61	96.64	97.69	95.61	96.64	99.56	97.44	98.49	97.69	95.61	96.64
**Micro**	97.80	94.22	**95.98**	97.80	94.23	95.98	99.17	95.54	97.32	97.80	94.23	95.98
**Macro**	97.35	96.33	**96.84**	97.35	96.33	96.84	99.25	98.21	98.72	97.35	96.33	96.84

**Table 11:** Detailed Performance Metrics: NER-Vietnamese-Electra-Base.

Tag	Strict			Partial			Entity Type			Exact		
	P	R	F1	P	R	F1	P	R	F1	P	R	F1
CQ	96.10	94.01	95.04	96.10	94.01	95.04	98.52	96.38	97.43	96.10	94.01	95.04
ĐT	96.63	90.67	93.55	96.63	90.67	93.55	99.18	93.06	96.03	96.63	90.67	93.55
VBPL	96.02	96.26	96.14	96.02	96.26	96.14	98.80	99.03	98.91	96.02	96.26	96.14
NG	99.98	99.98	99.98	99.98	99.98	99.98	99.98	99.98	99.98	99.98	99.98	99.98
SL	96.15	95.85	96.00	96.15	95.85	96.00	99.33	99.02	99.18	96.15	95.85	96.00
**Micro**	97.04	94.24	**95.62**	97.05	94.25	95.63	99.02	96.16	97.57	97.05	94.25	95.63
**Macro**	98.06	97.91	**97.99**	98.06	97.91	97.99	99.65	99.50	99.58	98.06	97.91	97.99

#### 5.7.1 Precision–recall trade-off analysis.

A consistent precision-dominant pattern emerges across all models and most entity classes. At the micro level, all architectures exhibit higher precision than recall: PhoBERT+CRF (*P* = 98.83, *R* = 97.09, gap = +1.74%), PhoBERT (+1.18%), Electra (+2.80%), and BiLSTM+CRF (+3.58%). This indicates that false negatives (missed entities) are systematically more prevalent than false positives (spurious predictions) across all architectures, a characteristic attributable to the complexity of Vietnamese administrative text where entities can be embedded in long, clause-heavy sentences.

The precision–recall balance varies substantially by entity class:

- *CQ (Agency)*: All models are precision-biased (P−R≥+2.09%), indicating that agency names, when predicted, are almost always correct, but some agencies embedded in complex administrative contexts are missed. PhoBERT+CRF achieves the best recall (*R* = 96.65) among all models while maintaining the highest precision (*P* = 98.92).
- *ĐT (Object)*: This class exhibits the most severe precision–recall imbalance, particularly for non-Transformer models. BiLSTM+CRF shows a + 7.38% gap (*P* = 97.35, *R* = 89.97), meaning it misses nearly 10% of Object entities—a critical failure for administrative workflows where identifying all relevant parties is essential. PhoBERT+CRF reduces this gap to +2.73% while raising precision to 98.55, demonstrating that contextual embeddings combined with structural constraints are necessary to resolve the semantic ambiguity inherent in this class.
- *VBPL (Legal Document)*: Uniquely among entity classes, VBPL shows a *recall-dominant* pattern for Transformer-based models: PhoBERT (P−R=−1.84%) and PhoBERT+CRF (−1.29%). This suggests that Transformers aggressively identify legal citation patterns but occasionally over-predict boundaries, generating false positives with slightly imprecise spans. The CRF layer partially corrects this by improving precision from 95.26 to 96.42 (+1.16%) while preserving high recall (97.71), demonstrating that the CRF’s transition constraints help tighten entity boundaries for structurally complex citations.
- *NG (Datetime)*: Near-perfect balance across all models (P−R<±0.5%), with F1 scores exceeding 99.7%. The highly formulaic nature of datetime expressions in administrative text makes this class trivially recognizable for all architectures.
- *SL (Quantity)*: Moderately precision-biased across all models. PhoBERT+CRF achieves the best F1 (97.67) with a balanced *P* = 98.15 and *R* = 97.20, while BiLSTM+CRF shows a larger gap (+2.08%), indicating difficulty recalling quantity expressions that deviate from common templates.

#### 5.7.2 Cross-model comparison by entity class.

Analysis of per-entity F1 variance across models reveals a clear difficulty hierarchy. ĐT (Object) is the most challenging class (F1 range: 93.51–97.17, σ=1.92), followed by VBPL (93.91–97.06, σ=1.34) and CQ (95.04–97.77, σ=1.27). In contrast, NG exhibits near-zero variance (σ=0.11), confirming that datetime recognition is effectively a solved problem for neural models on this corpus.

This difficulty hierarchy correlates with entity-specific linguistic properties:

- *ĐT* entities are semantically ambiguous—the same noun phrase can denote an administrative Object or a generic reference depending on context. Transformer models (PhoBERT: 96.45, PhoBERT+CRF: 97.17) substantially outperform non-contextual architectures (BiLSTM+CRF: 93.51, Electra: 93.55), confirming that deep contextual embeddings are essential for resolving this ambiguity. The + 3.66% gap between PhoBERT+CRF and BiLSTM+CRF on ĐT is the largest per-entity improvement observed across all class–model pairs.
- *VBPL* entities require both semantic understanding and structural pattern matching due to nested alphanumeric citation formats. Notably, XLM-RoBERTa (96.21%) outperforms BiLSTM+CRF (93.91%) by 2.30% on this class despite performing worse overall, suggesting that multilingual pre-training captures cross-lingual patterns in legal citation formats. PhoBERT+CRF (97.09%) leads all models on VBPL, with the CRF layer contributing a + 0.96% improvement over pure PhoBERT by enforcing valid citation boundary transitions.
- *CQ* entities are relatively well-defined (government agency names), yet still show a 2.73% F1 range across models (95.04–97.77), indicating that abbreviations and context-dependent agency references remain challenging for weaker architectures.

#### 5.7.3 Micro F1 vs. macro F1 divergence.

The relationship between Micro F1 and Macro F1 reveals important information about class-level equity across models. PhoBERT+CRF uniquely achieves Macro F1 (98.22%) *exceeding* Micro F1 (97.95%) by +0.27%, indicating that its performance is uniformly strong across all entity classes including minority ones. In contrast, Electra shows the largest Macro–Micro divergence (+2.37%), where Macro F1 (97.99%) substantially exceeds Micro F1 (95.63%). This pattern indicates that Electra achieves high per-class averages but struggles with the most frequent entity classes that dominate the micro-averaged score, likely due to its Replaced Token Detection pre-training objective being less effective for the domain-specific vocabulary of Vietnamese administrative text.

BiLSTM+CRF exhibits a Macro–Micro gap of +0.86%, consistent with its difficulty on the high-frequency ĐT class (F1: 93.51) which depresses the micro average. PhoBERT (Softmax) shows the most balanced profile (gap: −0.13%), with Macro F1 slightly below Micro F1, indicating marginally weaker performance on minority classes relative to frequent ones.

#### 5.7.4 CRF layer effect: Per-entity precision and recall decomposition.

Comparing PhoBERT (Table 8) with PhoBERT+CRF (Table 9) at the per-entity level reveals that the CRF layer’s contribution is not uniform but varies systematically by entity complexity:

- *Precision-driven gains on ĐT and VBPL*: For ĐT, the CRF improves precision by +1.58% (96.97→98.55) with minimal recall change (−0.11%), indicating that the CRF eliminates false positive Object predictions by enforcing valid transition sequences. For VBPL, precision improves by +1.16% (95.26→96.42) with recall also increasing (+0.61%), showing that the CRF simultaneously tightens boundaries and captures more complete citations.
- *Balanced gains on SL*: Both precision (+0.97%) and recall (+0.61%) improve for the Quantity class, suggesting the CRF helps with both boundary detection and entity completion for numerical expressions.
- *Marginal effect on NG*: The CRF produces negligible change (ΔF1 =−0.04%) on Datetime entities, which are already near-perfectly recognized by the base PhoBERT model (F1: 99.99). This confirms that structural constraints add value primarily for entities with complex or ambiguous boundary patterns, not for highly regular expressions.
- *Macro-level balance improvement*: The CRF layer shifts the Macro–Micro relationship from −0.13% (PhoBERT) to +0.28% (PhoBERT+CRF), indicating that the CRF disproportionately benefits weaker entity classes, thereby improving overall class equity.

### 5.8 Ablation Study: The impact of structural constraints

To verify the hypothesis that “probabilistic constraints are essential for normative domains,” we conducted a fine-grained ablation study comparing the pure PhoBERT (Softmax) against the PhoBERT-CRF (Hybrid). As detailed in Table 12, the inclusion of the CRF layer yielded statistically significant improvements for structurally complex entities.

- Resolving Nested Citations: The most notable gain was observed in the Legal Document (*VBPL*) class, which improved by 0.96% (*p* < 0.05 via McNemar’s test). Legal citations in Vietnam often exhibit nested structures (e.g., “Decree 34” nested within “Decree 34/2016/NĐ-CP”). The pure Transformer often fragmented these entities. The CRF layer, by modeling transition potentials (e.g., $P(I_{VBPL}|B_{VBPL})\gg P(B_{CQ}|B_{VBPL})$), successfully enforced boundary continuity, correcting these fragmentation errors.
- Robustness on Ambiguous Objects: The Object (ĐT) class, which refers to individuals or organizations participating in procedures, also saw a substantial gain (+0.78%). This indicates that the hybrid model is better at distinguishing specific administrative roles from generic nouns using global sequence optimization.
- Comparing PhoBERT (Table 8) with PhoBERT+CRF (Table 9) reveals further information:
  - Overall Gain: Adding the CRF layer improved the Strict F1-score from 97.55% to 97.95% (+0.40%).
  - Structural Correction: The primary value of the CRF layer is observed in the Strict vs. Entity Type metrics.
    - In the pure PhoBERT model, the gap between Strict F1 (97.55%) and Entity Type F1 (98.83%) is 1.28%. This indicates that the model often identifies the correct type of entity but fails to predict the exact boundaries.
    - In the PhoBERT+CRF model, this gap narrows to 0.77% (97.95% vs. 98.72%). The CRF layer effectively optimizes the state transitions, correcting boundary errors (e.g., ensuring a multi-token entity is not fragmented) and enforcing structural consistency.

**Table 12:** Ablation Analysis on Complex Entity Classes (Strict F1).

Entity Class	Definition	PhoBERT (Softmax)	PhoBERT-CRF (Hybrid)	Δ Improvement
VBPL	Legal Document	96.13	97.09	+0.96%
ĐT	Object	96.42	97.20	+0.78%
CQ	Agency	97.56	97.75	+0.19%

### 5.9 Computational efficiency and deployment feasibility

For e-Government applications, model accuracy must be balanced against inference latency. We measured the average processing time per sentence (ms/sent) on a standard server configuration: Intel Xeon Gold 6226R CPU (2.90GHz), 64GB RAM, Single-threaded inference, and Batch size = 1.

- Latency Analysis:
  - BiLSTM+CRF: 15.20 ms
  - XLM-R-Base: 46.8 ms
  - PhoBERT (Softmax): 42.50 ms
  - PhoBERT-CRF: 44.10 ms
- Trade-off Justification: The addition of the CRF decoding layer added a negligible overhead of ≈1.6 ms per sentence compared to the standard PhoBERT. Given the critical nature of legal accuracy—where a single missed citation can invalidate an administrative decision—the 0.96% accuracy gain on legal documents justifies this marginal increase in latency. The proposed framework remains well within the acceptable threshold for real-time document processing workflows (<50 ms).

## 6 Example analysis: Qualitative comparison of model outputs

To demonstrate the practical engineering advantages of the proposed Hybrid PhoBERT-CRF architecture over the baselines, we conducted a qualitative error analysis on complex administrative entities. This analysis validates our hypothesis: while the Transformer encoder captures semantic meaning, the CRF decoding layer is strictly required to enforce the boundary constraints of administrative entities. Table 13 presents a comparison of predictions generated by the baseline (PhoBERT) and the proposed framework (PhoBERT-CRF) on real-world samples from the PAP_NER test set.

**Table 13:** Qualitative Comparison of Structural Error Resolution (Color-Coded Analysis).

Case ID	Input Sentence Fragment	PhoBERT (Baseline)	PhoBERT-CRF	Engineering Analysis
**1. Boundary Truncation**	*Vietnamese:* “Hồ sơ nộp tại **Ủy ban nhân dân xã Đồng Văn** vào giờ hành chính.” *English:* “The dossier is submitted at the **People’s Committee of Dong Van Commune** during office hours.”	**[CQ]** Ủy ban nhân dân *(People’s Committee)* ✘ *Truncated Match*	**[CQ]** Ủy ban nhân dân **xã Đồng Văn** *(People’s Committee of Dong Van Commune)* *✓ Full Match*	**Fragmentation Error:** The baseline correctly identifies the semantic head “*Ủy ban nhân dân*” but **fails to capture the hierarchical suffix** “*xã Đồng Văn*”. The CRF layer **enforces continuity**, correctly capturing the full administrative unit.
**2. False Positive & Noise**	*Vietnamese:* “Văn bản số **123/BC-UBND** ngày 15/10/2023 của **Ủy ban nhân dân tỉnh**.” *English:* “Document No. **123/BC-UBND** dated 15/10/2023 of the **Provincial People’s Committee**.”	**[VBPL]** BC-UBND ✘ *Hallucination* **[CQ]** Ủy ban nhân dân *(People’s Committee)* ✘ *Truncated Match*	**[O]** 123/BC-UBND ***✓*** *Correct Suppression* **[CQ]** Ủy ban nhân dân **tỉnh** *(Provincial People’s Committee)* ***✓*** *Full Match*	**Structural Correction:** The baseline **hallucinates a Legal Document ([VBPL])** tag for a generic document number (“BC-UBND”). The CRF correctly **suppresses the invalid citation pattern** and extends the Agency boundary to include “Provincial” (*tỉnh*).

### 6.1 Analysis of boundary truncation (agency entities)

In Case 1, the input text refers to a specific local authority: “Ủy ban nhân dân xã Đồng Văn” (People’s Committee of Dong Van Commune).

- The Baseline Failure: The pure PhoBERT model utilizes token-level classification. Semantically, “Ủy ban nhân dân” (People’s Committee) is a strong signal for the Agency (CQ) class. However, the suffix “xã Đồng Văn” (Dong Van Commune) contains proper nouns and common nouns that often appear in non-entity contexts. Consequently, the Softmax classifier prematurely terminates the entity at “dân” (People), resulting in a Truncation Error. In a digital government routing system, this is a critical failure, as it routes the document to a generic category rather than the specific local commune.
- The Hybrid Solution: The PhoBERT-CRF model successfully captures the full span. This is achieved because the CRF layer models the transition probability $A_{y_{i-1},y_{i}}$. The model learns that within the administrative domain, the transition probability remains high even when the token itself (e.g., “xã” / Commune) is semantically ambiguous. The global score optimization ensures the “Agency” tag covers the entire hierarchical string.

### 6.2 Analysis of false positives (noisy citations)

In Case 2, the text contains a document code “123/BC-UBND,” which resembles a legal citation but is effectively a generic reference number (noise).

- The Baseline Failure: The PhoBERT model, reacting to local features like capitalized letters and hyphens, incorrectly labels “BC-UBND” as a Legal Document (VBPL) with moderate confidence (0.77). This represents a False Positive, which introduces noise into the downstream knowledge graph.
- The Hybrid Solution: The PhoBERT-CRF model correctly assigns the O (Outside) tag. The CRF structure imposes a penalty on citation formats that do not strictly adhere to learned legal templates (e.g., Number/Year/Type-Agency). By optimizing the sequence probability over the entire sentence, the CRF determines that this token sequence does not fit the valid transition structure of a VBPL entity, effectively filtering out administrative noise.

These examples confirm that the performance gains reported in Section 5 (e.g., + 0.96% on VBPL) are driven by the CRF’s ability to repair structural defects—specifically boundary fragmentation and pattern non-conformity—that semantic embeddings alone cannot resolve.

## 7 Discussion

This section discusses the key findings and implications of our experimental results. We first analyze the interplay between semantic and structural modeling (Section 7.1), then examine the advantages of language-specific models (Section 7.2), followed by a discussion of dataset quality (Section 7.3), broader implications for Vietnamese administrative NLP (Section 7.4), and current limitations (Section 7.5).

### 7.1 Semantic and structural modeling in administrative NER

The superior performance of the PhoBERT-CRF framework (97.95%) validates our core engineering hypothesis: administrative texts require a dual-engine approach. While PhoBERT serves as a powerful Semantic Engine, capturing deep contextual cues to distinguish ambiguous entities (e.g., distinguishing an Object from an Agency based on sentence role), it fundamentally lacks the mechanism to enforce valid output sequences. By integrating the CRF as a Structural Engine, we mathematically enforced the “grammar” of administrative procedures. This synergy explains the statistically significant gain (+0.96%, *p* < 0.05) in the Legal Document (VBPL) class. In pure Transformer architectures, the complex alphanumeric patterns of legal citations often led to fragmentation errors. The CRF layer corrected these by learning high transition potentials ($A_{y_{i-1},y_{i}}$) for intra-entity tokens, effectively “gluing” the fragmented components back together. For e-Government systems, where a single broken citation link can invalidate a workflow, this structural robustness is non-negotiable.

### 7.2 Language-specific models in specialized domains

PhoBERT’s outperformance of multilingual XLM-RoBERTa (97.51% vs. 95.43%) despite being half the size indicates that for specialized low-resource domains, language-specific optimization is more valuable than raw model capacity. This finding has implications for other low-resource languages requiring domain-specific NLP systems.

### 7.3 Dataset quality and annotation methodology

The strong Inter-Annotator Agreement (κ=0.85) and high performance across all models (minimum 95.43% baseline) indicate PAP_NER is a high-quality, well-defined benchmark. Source-stratified splitting ensures evaluation measures true generalization rather than template memorization. The convergence between Strict and Partial F1 scores suggests errors are primarily complete misses rather than boundary overlaps.

### 7.4 Broader implications and transferability

This work establishes a foundational dataset and benchmark for Vietnamese administrative NLP. The benchmarked PhoBERT-CRF configuration provides a reproducible baseline for future research, and public release under the Creative Commons BY 4.0 license promotes community-driven advancement of Vietnamese NLP.

Although PAP_NER and the trained models target Vietnamese administrative text, several outcomes of this study transfer beyond that setting. First, the corpus-construction pipeline—rule-based and LLM-assisted pre-annotation with local PII masking, MinHash near-duplicate removal, and source-stratified (group-aware) splitting—is language- and domain-agnostic; it offers a cost-effective recipe for building gold-standard NER resources in other low-resource languages and in other normative domains such as legal, clinical, or financial-compliance text, where templated phrasing and long structured spans pose similar annotation challenges. Second, our central empirical finding—that a dedicated monolingual encoder outperforms a larger multilingual one on a specialized low-resource domain—is consistent with results reported for other underrepresented languages [23,24] and offers a practical heuristic for practitioners selecting encoders under data-sovereignty and memory constraints. Third, the deployment-aware benchmarking protocol, which jointly weighs accuracy, latency, and footprint, is readily portable to other national e-Government pipelines facing comparable operational limits. Realizing this transfer in a new language or domain still requires fresh annotation and fine-tuning, since the structural patterns learned by the CRF and the lexical coverage of the encoder are specific to the source text (Section 7.5); the methodology, however, carries over directly.

### 7.5 Limitations and engineering trade-offs

While effective, the proposed framework entails specific trade-offs:

1. *Linguistic Scope*: Model is optimized for formal administrative text; performance may degrade on informal language where templates are absent.
2. *Geographic Coverage*: Four provinces provide regional diversity; comprehensive coverage of all 28 provinces would strengthen generalization claims.
3. *Class Imbalance*: Quantity (SL) class underrepresented (7.2%); transfer learning to SL-heavy domains may require additional fine-tuning.
4. *BIO Tagging Limitation*: The BIO (Begin, Inside, Outside) tagging scheme adopted in this work does not natively support nested or discontinuous entities. While legal citations may contain nested sub-components (e.g., a decree number nested within a full citation span), our scheme treats each entity as a single contiguous span. We selected BIO for compatibility with standard NER frameworks and prior Vietnamese NER benchmarks (VLSP 2016–2021). Future work should explore span-based or nested NER approaches [32] for extracting fine-grained sub-components within legal citation entities.

## 8 Conclusion and future work

This study addresses the critical infrastructure gap in Vietnamese government NLP by introducing PAP_NER, the first large-scale, gold-standard administrative NER corpus. Key contributions include:

- Dataset: We introduced PAP_NER, a corpus of 162k sentences. This represents the first large-scale, gold-standard “material” specifically engineered for Vietnamese e-Government workflows, resolving the data scarcity issue that has historically impeded research in this domain.
- Methodology: Comprehensive benchmarking of five neural architectures with statistical significance testing establishing performance baselines.
- Empirical Evidence: Demonstration that Vietnamese administrative NER benefits from hybrid architectures coupling semantic understanding with structural constraints.
- Reproducibility: Complete experimental documentation and public dataset release enable community validation and extension

The hybrid PhoBERT-CRF framework achieves 97.95% F1 with statistically significant improvements on complex nested entities (+0.96%, p < 0.05). This work provides a foundational resource for Vietnamese administrative NLP and demonstrates methodology applicable to domain-specific NER in other low-resource languages. Our future research will focus on extending the lifecycle of this framework in three directions:

- Model compression for edge computing: We aim to apply Knowledge Distillation to compress the heavy PhoBERT-CRF model into a lighter student network (e.g., DistilPhoBERT). This will reduce inference latency to sub-20ms levels, enabling deployment on resource-constrained edge devices used by field officers.
- Handling informal citizen queries: Currently, our model is optimized for formal administrative documents. We plan to explore domain adaptation techniques to extend the model’s robustness to informal, non-standard text found in e-Government chatbots and citizen feedback channels, where strict syntactic templates are often absent.
- Nested entity recognition for legal sub-components: We aim to investigate span-based and nested NER architectures that can extract fine-grained sub-components within legal citations (e.g., decree type, number, year, issuing agency) as individual entities, enabling richer knowledge graph construction for legal document management systems.
